# Introducing the North Water: Histories of exploration, ice dynamics, living resources, and human settlement in the Thule Region

**DOI:** 10.1007/s13280-018-1030-2

**Published:** 2018-03-07

**Authors:** Kirsten Hastrup, Anders Mosbech, Bjarne Grønnow

**Affiliations:** 1 Department of Anthropology, University of Copenhagen, Øster Farimagsgade 5, 1353 Copenhagen K, Denmark; 2 Department of Bioscience, Arctic Research Centre, Aarhus University, Frederiksborgvej 399, 4000 Roskilde, Denmark; 3 The National Museum of Denmark, Frederiksholms Kanal 12, 1220 Copenhagen K, Denmark

**Keywords:** Arctic exploration, Human migration, Ice dynamics, Living resources, Sea–ice community, Thule region

## Abstract

The North Water is a recurrent polynya in the High Arctic situated between Northwest Greenland and Ellesmere Island of Canada. The North Water makes a dynamic space, where various processes may enhance or obstruct each other, accelerating or halting particular modes of human–animal relations in the region, where life itself depends on the North Water. This will be discussed in four steps. The first step posits the North Water as a perceived oasis for explorers and whalers hailing from Europe or America in the nineteenth century. The second step concentrates on the diverse rhythms inherent in the ice conditions, as affected by trends that are set in motion elsewhere. The third step highlights the implications of the dynamics of the ice and sea currents for animal life in the region. The fourth step gives an overview of human settlement patterns around the North Water across the ages. The article shows how natural and social features are deeply implicated in each other, even if they are not directly co-variant.

## Introduction

**Fig. 1:**
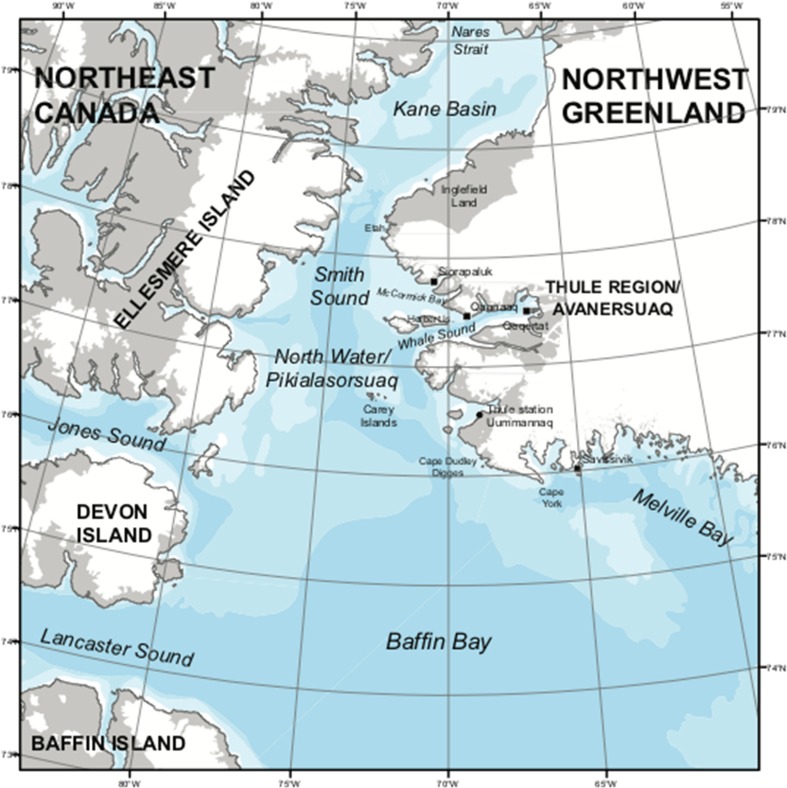
Map of the North Water region (drafted by Kasper Lambert Johansen)

The central figure in this Special Issue is the North Water (Pikialasorsuaq), a recurrent High Arctic polynya at the top of Baffin Bay between Northwest Greenland (Avanersuaq) and Ellesmere Island and Baffin Island on the Canadian coast, roughly covering the area between 76°N to 79°N and 70°W to 80°W at its peak (Fig. 1). While itself an open water area, the North Water is demarcated by the surrounding, ever shifting sea–ice most of the year, and subject to seasonal changes in wind and sea currents as well as changes in temperature. The polynya responds to these factors; it expands or contracts and caters for different life forms at different times; it is these dynamics, including the present global warming, that cause destabilization of ice edges at seasons where they used to be stable. This is also what makes us see the North Water as a forceful agent in the entire region, and which we shall introduce here as a key to the interplay between ice conditions, animal migrations, human settlements, and hunting opportunities in both short- and longer term perspectives.

The Thule region is named from the Thule trading station that was established in 1910 at the ancient site of Uummannaq, at the heart of the region’s hunting grounds. Later, in the 1920s, the name Thule was given also to a particular prehistoric culture, the Thule Culture, remains of which were found in an ancient kitchen midden at Uummannaq. In its initial phase around 1200–1400 ad, this culture based its subsistence economy on hunting of bowhead whale (*Balaena mysticetus*), walrus (*Odobenus rosmarus rosmarus*), and seals, especially ringed seal (*Phoca hispida*), by means of sophisticated tools and technologies, including whaling harpoons and *umiaat* (large skin boats). The Inuit of the Thule Culture also introduced dog sledges and kayaks for hunting and transportation (McGhee 1997). The combination of these technologies and a social organization that facilitated communal hunting of big game was new to Greenland and reflects a novel way of exploiting the living resources of the polynya in comparison with the earlier cultures of the High Arctic. The people of the Thule Culture are the ancestors of the present Inughuit population in the area, currently numbering about 750 people (Statistics Greenland 2017).

## Histories of western discovery: Arriving at the north water

The passage to the North Water invariably goes through the Davis Strait, named after John Davis, the first navigator sent out in search of a Northwest Passage in late sixteenth century under the reign of Elizabeth I. In his third failed attempt, he made it as far as somewhere between 70° and 73°N before he had to return south along the western coast of the Strait due to foul weather. Today, the Davis Strait is geographically defined by the 70°N parallel between Greenland and Baffin Island and the 60°N parallel between Greenland and Labrador.

The charts and observations made by Davis attracted European whalers and alerted a new generation of explorers to search for the (as yet) unknown passage towards the northwest. One of them was William Baffin, who in the period 1612–1616 made five expeditions in this pursuit, without locating a passage but discovering so much else. Clements Markham, who edited and published Baffin’s journals for the Hakluyt Society in 1881, wrote:

The fame of Baffin mainly rests upon the discovery of the great bay extending north from Davis Strait. Passing Hope Sanderson, the furthest point reached by Davis, Baffin came to the Women Islands, and the Baffin Islands off Cape Schackleton, at the southern end of Melville Bay. He then crossed Melville Bay, between the 1st and 3rd of July, a most extraordinary piece of good fortune; and, arriving off Cape Dudley Digges, he entered the *North Water*, which “anew revived our hope of a passage”. (Markham 1881: LII)

While by the nineteenth century, the name of the North Water clearly had gained growing recognition, it was not yet in use at Baffin’s time; he simply speaks of coming into “an open sea” on the 1st of July at 75°40′N (Baffin in Markham 1881, p. 144). Baffin pressed farther north while the opportunity lasted, finding and naming several sounds, islands, and capes along the way, names of which many are still in use (by foreigners). On the fourth day, they were forced to seek shelter in a little cove in a great sound, until the southwest wind abated. Baffin wrote:

In the Sound we saw great numbers of whales, therefore we called it Whale Sound, and doubtless, if we had been provided for killing of them, we might have stroke very many. It lies at the latitude 77°30′. (Baffin in Markham 1881: 145)

It is worth noting that although Baffin does not actually name the North Water, he certainly found it and noted the abundance of game in Smith Sound (named by him). Apart from the whales, he explicitly mentions the walrus and the sea-unicorn [narwhal, *Monodon monoceros*)]. Nowhere did he find signs of any people, apart from the northernmost Greenlanders he met before crossing the Melville Bay in the (present) Upernavik District, at the so-called Women Islands. The key point is that Baffin certainly had found the North Water, if not yet named.

The next European explorer known for his travel in the North Water region is Sir John Ross, who was sent out by the British Admiralty in 1818 to find a passage out of Baffin’s Bay, as it was now called. Ross quoted extensively from his official instructions, providing us with the state of general knowledge about the North Water region at the time.

From the best information we have been able to obtain, it would appear that a current of some force runs from the northward towards the upper part of Davis’ Strait, during the summer season, and perhaps, for some part of the winter also, bringing with it fields of ice in the spring, and ice bergs in the summer.

This current, if it be considerable, can scarcely be altogether supplied by streams from the land, or the melting of ice; there would, therefore, seem reason to suppose, that it may be derived from an open sea; in which case, Baffin’s Bay cannot be bounded by land, as our charts generally represent it, but must communicate with the Arctic Ocean. (Instruction, in Ross 1819: 3–4)

The Admiralty had obtained their information from several masters of whaling vessels, and what they were really looking for was a passage to the west somewhere at the top of Baffin’s Bay. Although Ross made it from the Melville Bay—so named by himself in honour of his patron in the Admiralty (Ross 1819, p. 67) to roughly 77°N, he did not find any passage, but confirmed that the bay was closed at the top. Sensationally, Ross was the first European to meet and describe people living on the shores of Cape York north of the Melville Bay. He called them the Arctic Highlanders, and noted how they believed themselves to be alone in the universe (Ross 1819, p. 124).

Yet the British Admiralty did not give up; they recognized the currents from the North—partly accounting for the North Water, as we now know. Several new attempts were made during the following decades to find what had to be there—an opening towards the northwest. Among the searchers was John Franklin, who disappeared during the attempt, which again spurred numerous rescue expeditions. One of these was the expedition of Elisha Kent Kane in 1853–1855. His primary objective was to search for the Franklin expedition with a secondary goal to gain more knowledge about the possibilities for further passage towards the northwest. Kane had read Ross and others and noted their dread in passing Melville Bay, but in his case it went relatively smoothly: “Midnight. – We are clear of the bay and its myriad of discouragements. The North Water, our highway to Smith’s Sound, is fairly ahead” (Kane 1856, I: 39).

Here, finally, the North Water is named. Kane reports in more detail on the character of ice and water, and he gives a small note on the North Water as such: “The North Water, although its position varies with the character and period of the season, may be found, under ordinary conditions, in the month of August at Cape York” (Kane 1856, p. 454). The salient feature of the North Water in Kane’s description is its openness, making passage possible, or—in his own phrasing—providing a highway into the region. Isaac I. Hayes, who had taken part in Kane’s expedition, visited the region again on his own account (1860–1861) and wintered in the same general area as Kane, in the Foulke Fjord, near Etah. The North Water had become a well-established fact by then, Hayes simply noting after having traversed the Melville Bay that “we are now in the North Water” (Hayes 1866, p. 65).

Like his predecessors in the region, Robert Peary—whose dream was not to find the Northwest Passage but to reach the North Pole, and who sojourned in the Thule region on and off from 1891 to 1909—was “baffled by the ice of Melville Bay” (Peary 1898, I: 62). He noted his relief when finally entering into the open waters, making it possible to travel swiftly to his destination in McCormick Bay (as it happened). During a December moon, Peary and some men went south to Cape York on sledge to map the prominent points on the coast, “but the frozen condensation from the North Water, which was steaming like a huge black cauldron, shrouded the coast in a silvery veil, and rendered the stars invisible most of the time” (Peary 1898, II: 327). Even in the winter night, the open water made itself acutely felt.

Generally, after Baffin, the North Water took shape as a distant goal worth pursuing, either in the interest of whaling during the short period of accessibility every year or in the hope of finding a passage to shores of more important economic gains. The open water, as yet of unknown constitution, lured people to the North, just as it had made it possible for the prehistoric Thule Inuit to settle there and for their descendants to remain. In its own way, the North Water made a multitude of histories (Hastrup 2016).

## Ice dynamics: Stable currents and volatile edges

The North Water is defined in relation to the ice, if not *with* the ice (Fig. 1). With air reconnaissance and satellite data, it became possible to chart the annual cycle with some precision in the 1960s (Dunbar and Dunbar 1972), even though winter darkness still prevented permanent observation. Adding to this difficulty is the invariable lack of distinctiveness, even disappearance, of the North Water in summer, when it opens up and connects Kane Bassin with Baffin Bay. This takes us to the dynamics of the surrounding ice as a decisive factor in the North Water’s constitution.

Today, satellite-borne sensors provide more comprehensive information on the extent of the open water, and it is clear that it evolves seasonally from a relatively small area in winter, often covered by a thin layer of new ice in shifting places, to a larger area of ice-free water in June, while in July–August it ceases to exist as a distinct ice-bounded region. The existence of the North Water is due to several factors: An ice arch is formed across the narrowest part of the Nares Strait blocking the ice flow from the north. This allows the northerly winds channelled through the strait as well as the Baffin Current to remove new ice formed through high rates of surface freezing south of the arch. Thus, the North Water is a region with high rates of heat exchange (Barber et al. 2001, p. 344; Bâcle et al. 2002, p. 4923). In the 1960s when aerial surveys began, it was ascertained that the northern ice arch would usually break up in late July or August. “Once the bridge has gone it becomes impossible to define the North Water at all from ice observations, as the area becomes merely part of the southward drift route, or melting ground, for ice from the Kane Basin and farther north” (Dunbar 1969, p. 441). Since the late 1990s, the formation of the Ice arch is more unreliable and the Ice arch seems to break up earlier (Marchese et al. 2017)

The opening of the North Water is also related to the relatively warm water upwelling of the West Greenland Current (WGC) near the west coast of Greenland (Marchese et al. 2017), which brings warm water to the base of the surface mixed layer that may be entrained by convection particularly in the autumn. This slows ice growth on the Greenland side of the polynya and results in thinner ice (Melling et al. 2001; Barber and Massom 2007). From the opposite direction, the North Water region is fed by an inflow of cold water from the Arctic Ocean flowing southwards through Smith Sound, meeting the warmer influx from the West Greenland Current (Melling et al. 2001, p. 302). The warm inflow from the West Greenland Current flows as far north as 77°N, hitting the Carey Islands and nearly stopping there due to shallow water, yet occasionally pressing a bit further along the eastern shores of the islands (Ingram et al. 2002, p. 4900). Worth noting in connection with the West Greenland Current are the small amounts of Siberian driftwood that occasionally reach the shores of Thule from South Greenland carried there by the East Greenland Current (Hellmann et al. 2013).

At the level of human experience, the combined effects of the dynamics of the North Water and the present global warming are acutely felt. Since the turn of the century, the ice conditions have changed remarkably and left the hunters to speculate about a future without the well-known seasonal changes and hunting opportunities. The ice edge is moving, widening the opening of the North Water at unexpected times and closing down access to traditional hunting grounds; in the process, new knowledge is assembled, but projections for the future seem short-lived (Hastrup 2013). As a hunter said a few years back:

The ice has changed. Right here, it is less than half a meter [he shows about 30 centimetres by his hands]. There are so many currents now around Herbert Island, which makes the ice thin close to land. Between Herbert Island and Qaanaaq, I think the ice is between one metre and one and a half metre. There are no problems there, but here it is bad. It is difficult, but we know how to move. The water has opened up, and has therefore become warmer, and this means that ice melts from below. This is also due to new and warmer currents. So the ice is very thin and unsafe in many places. (Conversation with hunter, May 2010)

It is the thinned ice, which increasingly troubles the hunt in what used to be the annual peak of abundance thanks to the relatively stable ice edge. With the less stable polynya, the meaning of ice is changing (Gearheard et al. 2013).

## Living resources: Animal life by the north water

**Fig. 2:**
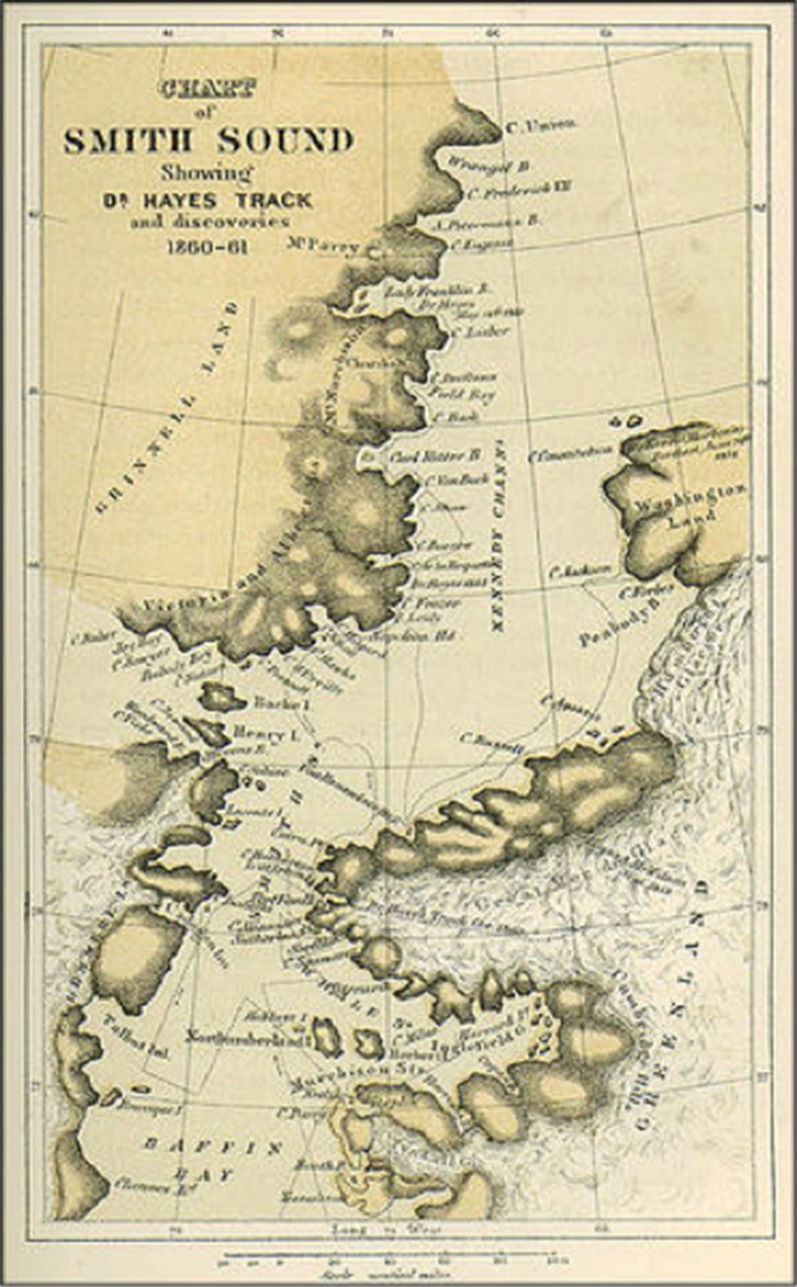
Map of Smith Sound published by Hayes (1866). Hayes wintered in Foulke Fjord (1860–1861) at the narrowest point of the Smith Sound, where he was stopped by the Ice. His tracks south of this relate to sailing, while north of this the tracks refer to sledging expeditions

In an attempt to circumvent the inaccuracies of aerial census techniques in measuring the relative abundance of wildlife in the region, a ground-based survey was made in the early 1990s on the coast of Ellesmere Island (France and Sharp 1992). Members of the expedition skied along the coast and counted what they saw; as it turned out, the method was not free of bias, nor was it necessarily more precise in its estimates, but it is interesting in its approach to ecological integrity, as France and Sharp call it. This refers to the wholeness, diversity, and degree of connectiveness within a biotic community, and can be viewed as an emergent property of ecosystems (France and Sharp 1992, p. 444). Inadvertently, this is a parallel to the way in which the Inughuit hunters perceive their surroundings—as a totality of diverse resources at particular points in time. A newer word for such ecological integrity is sea–ice community. This embraces all organisms from phytoplankton and upwards to higher trophic levels, seabirds, seals, whales, polar bears, and humans—all of them part of the biotic community and the ecosystem that depends on the ice *and* the open water in its midst, and crucially interdependent (AMAP 2011, pp. 59–61; Meltofte 2013, pp. 486–527; CAFF 2017) (Fig. 2).

The North Water has been presented as not only one of the largest polynyas but also one of the most productive ecosystems within the Arctic Circle (Stirling 1980). The understanding of the North Water (or NOW) polynya as an ecosystem took a major leap forward with the International North Water Polynya Study 1997–1999 (Deming et al. 2002). The study was undertaken as a combined icebreaker/ice-camp endeavour over a 3-year period. In 1998, there was a continuous 5-month spring and summer research effort, and in 1999 the research captured the late-summer features and fall closing of the polynya. The large number of interdisciplinary oceanographic and ecological studies spurred a huge number of publications including a special issue of *Deep Water Research II* (vol. 49, 2002) also presenting a clear picture of the productivity:

The North Water exceeded expectations in its ability to support high levels of primary production (monthly maximum of 5.3 g C m^−2^ day^−1^ observed near Greenland in May; Klein et al. 2002) and of new production (whole-polynya maximum of 1.7 g C m^−2^ day^−1^, again in May; Tremblay et al. 2002) over an unprecedented period of time for a High Arctic ecosystem (from March to October, north of 76°N; Odate et al. 2002; Klein et al. 2002). (Deming et al. 2002: 4888)

**Fig. 3:**
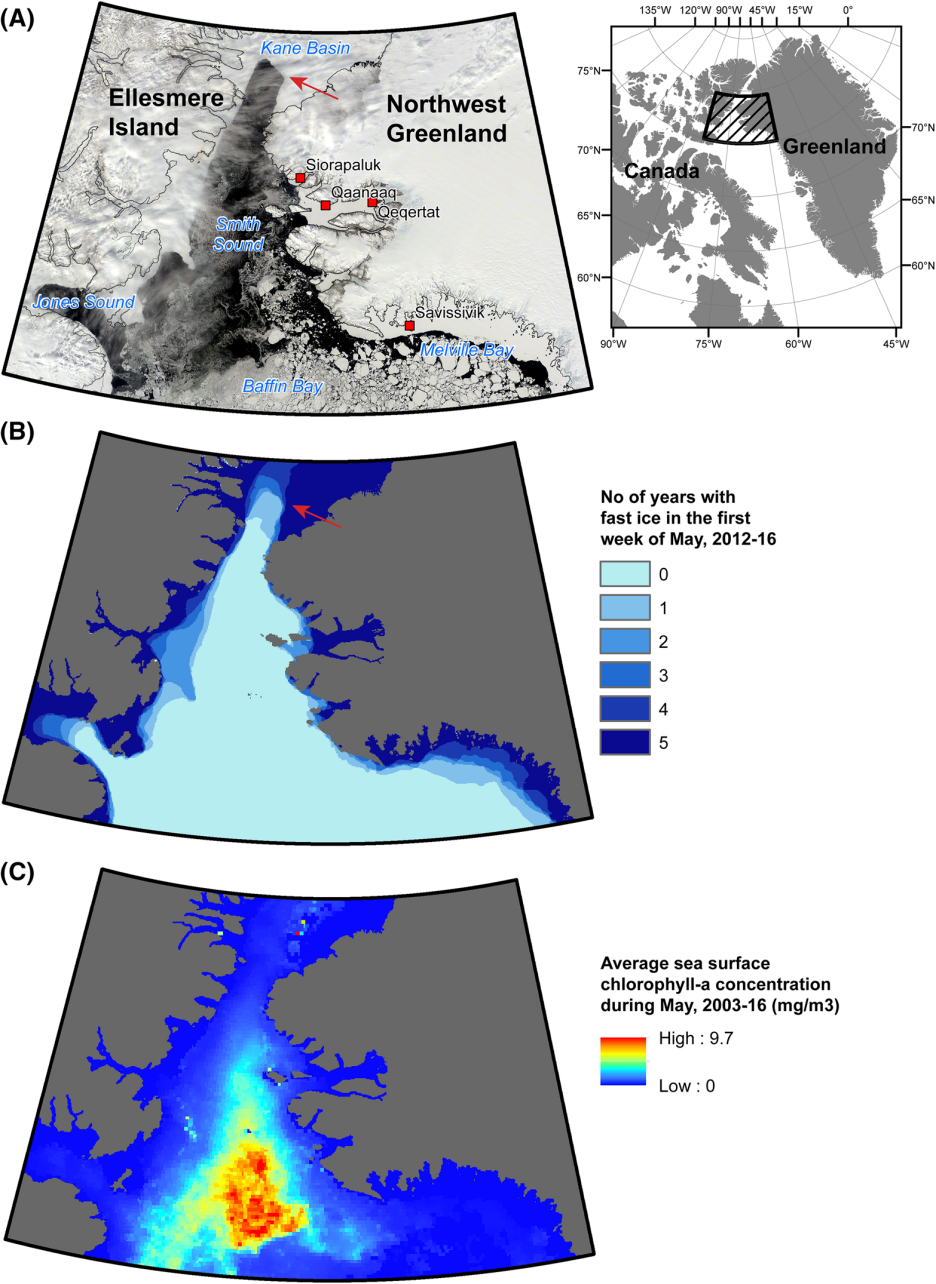
The North Water ice dynamics. **a** Satellite image of the North Water on 18 May 2015 [Terra MODIS (https://worldview.earthdata.nasa.gov)]. The North Water polynya is circumscribed by sea ice: to the north, east and west by land-fast ice, and to the south by drifting pack ice in the Baffin Bay. **b** Overlay analysis of the distribution of land-fast ice during the first week of May 2012–2016, based on data from Canadian Ice Service (http://nsidc.org/data/G02171). Notice the ice arch (arrow) in the southern part of Kane Basin, and how the land-fast ice defines all but the southern perimeter of the North Water. The colour scale indicates how many of the 5 years a particular area was covered by fast ice, highlighting stability/dynamics in the delineation of the North Water at this time of the year. The ice arch blocks the inflow of ice from the north, and the strong northern winds that prevail during winter tend to sweep the area south of the ice arch free from the new ice that continuously forms on the sea surface. **c** Average sea surface chlorophyll-a concentration in May 2003–2016 from Aqua MODIS (OCx Algorithm, 4 km resolution; https://oceancolor.gsfc.nasa.gov/cgi/l3). It can be seen how the North Water Polynya defines the spatial extent of the phytoplankton bloom at this time of the year. A minor component of the bloom is under the ice and not visible. The combination of open water, which allows sunlight to penetrate into the water, and a steady supply of nutrients to the upper water column, from deep winter mixing and especially late in the season in the eastern part also from the West Greenland Current, results in a strong primary production, which starts early in the spring and is maintained throughout most of the summer. Drafted by Kasper Lambert Johansen

The phytoplankton bloom starts on the Greenland side (in April), where the high phytoplankton biomass coincides with the earlier melt of the thinner ice (Fig. 3). On the Ellesmere Island side the bloom starts about two months later and the difference is evened out only in July. A new increase in phytoplankton is seen at the Greenland side in August with the entrainment of the West Greenland Current to the surface mixed layer, while the production on the western side stagnates and declines (Odate et al. 2002; Klein et al. 2002; Barber and Massom 2007).

**Fig. 4:**
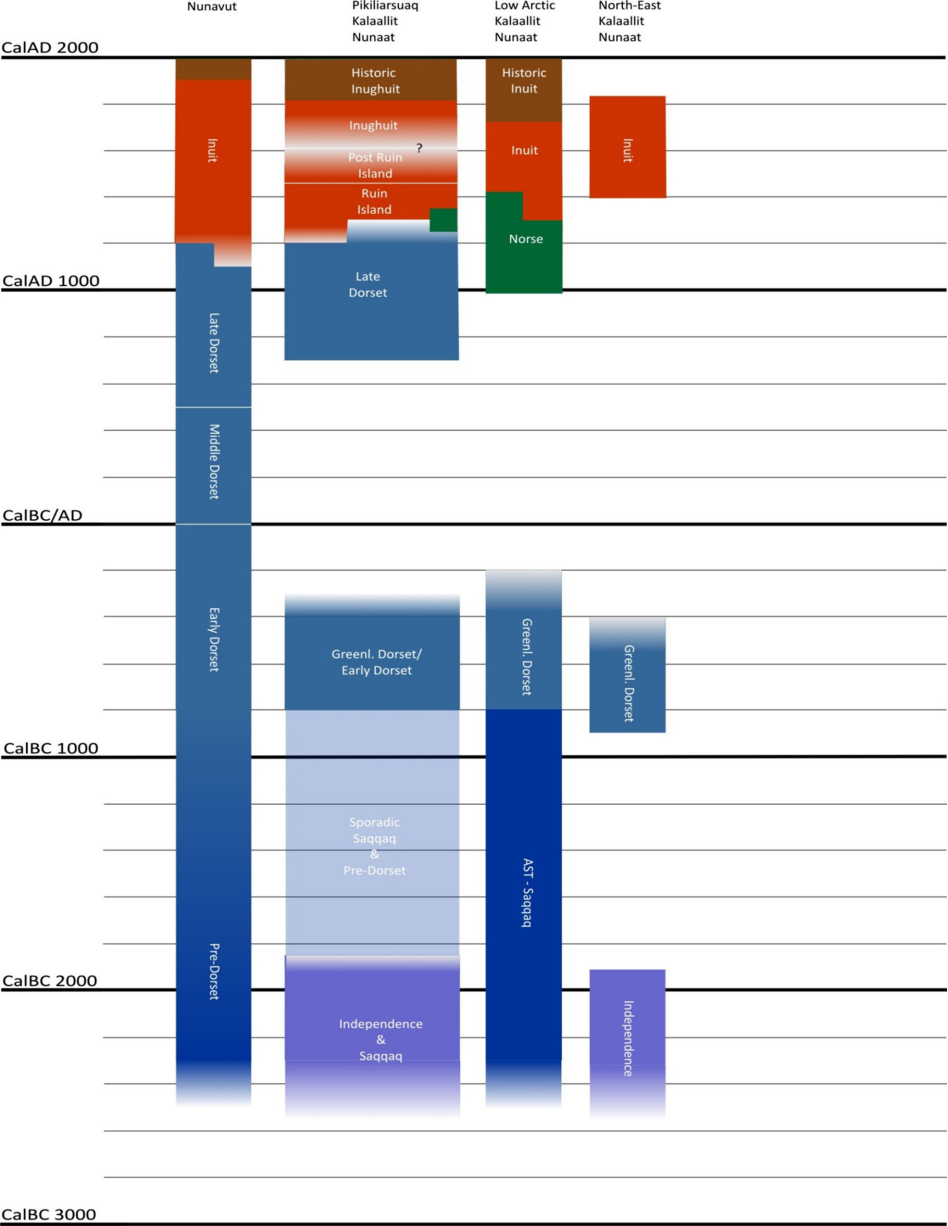
A general outline of the cultural chronology of Nunavut (Eastern Canadian Arctic) and Kalaallit Nunaat (Greenland), divided into Pikialasorsuaq (The North Water area), Low Arctic Greenland, and North-East Greenland. Redrawn by M. Appelt from the chart in Appelt, Friesen and Grønnow in Raghavan et al. (2014)

The large prolonged phytoplankton production sets the baseline for the reproductive success of the herbivorous copepods (Ringuette et al. 2002). These large Arctic herbivorous copepods (*Calanus hyperboreus* and *Calanus glacialis*) build lipid deposits during summer and are key species at the bottom of the marine food chain, while the pelagic amphipod *Themisto libellula* and polar cod (*Boreogadus saida*) are their key predators next in the food chain and lay the foundation for the food web sustaining the large marine mammal populations so characteristic for the NOW and so important for the hunters (Hobson et al. 2002; Tremblay et al. 2006; Heide-Jørgensen et al. 2013a, b, 2016; Dietz et al. 2018 for an overview of the hunting bag). The copepods also serve as a primary food source for little auks (*Alle alle*) (Karnovsky and Hunt 2002; Karnovsky et al. 2008). The small seabirds and their eggs are a basic resource for glaucous gulls (*Larus hyperboreus*) and Arctic fox (*Vulpes lagopus*), and their organic waste on the talus slopes where they breed fertilizes the ground, which in turn is the precondition for the lush vegetation on and in particular at the foot of the talus slopes. Here, populations of Arctic hare (*Lepus arcticus*) and muskox (*Ovibos moschatus*) feed on the plants. In short, the Thule region is a quite special region of the High Arctic because of the productive NOW marine ecosystem. An analysis of just one species, the little auk, and its ecosystem services to the entire community illustrates the depth of this claim (Mosbech et al. 2018) (Fig. 4).

Beyond the North Water and its shifting ice edges, other factors of ice and climate also influence animal life in the region, even if it is originating elsewhere. Christian Vibe (1967) pioneered with a hypothesis on how different drift-ice stages originating in the East Greenland Current (EGC) had great impact on west and northwest Greenland by way of the West Greenland Current (WGC) reaching up to the North Water. Vibe concentrated on historical periods, for which records of the hunt and the population were available. He identified three major periods, for which we shall mention only the consequences in the northwest, that is the Thule region: first, *the drift*-*ice stagnation stage* (c. 1810–1860), where the WGC did not reach very far north on the west coast, and where the weather in the northwest remained relatively cold, dry, and stable. This favoured the reindeer (*Rangifer tarandus*) in the region. Second, *the drift*-*ice pulsation stage* (c. 1860–1910), where the WGC (and hence the drift ice from the EGC) reached further north making the winters more wet and creating severe problems for muskox and reindeer, and for the Arctic fox. Third, there is *the drift*-*ice melting stage* (c. 1910–1960), where the ice from the ECG was strongly reduced along the western coast. This made the populations of marine mammals, foxes, and seabirds increase in the northwest according to Vibe’s hypothesis (1967).

The salient feature of Vibe’s pioneering work is the documentation of a certain “pulse” or oscillation on a multi-decadal scale in the climate, affecting animal presence of the Thule region through currents and drift ice originating in distant places. More recent research has focused on regional decadal climate oscillations (North Atlantic Oscillation [NAO] and Arctic Oscillation [AO] indices), which fluctuate between a positive and negative state every two or three decades and linked these climate fluctuations to animal population changes (Post and Forchammer 2002; Irons et al. 2008). It is not a simple causal relation, and the hunters’ prey is far from a fixed presence in the region. In spite of some regularity, it is affected by forces deriving from far beyond the polynya ecosystem. Together this influences animal patterns of movement and settlement in the Thule region (Hastrup et al. 2018).

Following the International North Water Polynya Study (1997–1999), there has been an oceanographic North Water initiative focused on a time-series approach at an decadal scale, which hopefully can give further understanding of the ecological change now exacerbated with the accelerating arctic climate change (Marchese et al. 2017). Some years ago, scientists suggested that because polynyas are so biologically and historically important, “their candidacy as World Heritage Sites… may be one of the means of ensuring their continual preservation in a pristine condition” (France and Sharp 1992, p. 445). We are now long past any vision of a pristine condition, let alone a clean environment. Not only is the biotic community in the North Water changing in response to the global warming, it is also subject to increasing contamination from mercury and new persistent organic pollutants, deriving from distant sources and resulting in severe health concerns among people in the Thule region (Dietz et al. 2018).

The uniqueness of the NOW ecosystem and the need for careful management is certainly acknowledged both internationally, e.g. in a recent workshop on potential candidates for marine World Heritage Sites (Speer et al. 2017), and nationally, e.g. in the *Arctic Strategy for the Kingdom of Denmark 2011*–*2020* (Anon. 2011), and in the identification of ecologically and biologically significant areas in Canada’s eastern Arctic (Fisheries and Oceans Canada 2015). We would like to stress that a protection initiative would also, hopefully, protect the hunt that is still basic to human life in this High Arctic region and can be considered part of a social-ecological system. Local involvement is important and principles from ecosystem-based and adaptive management could be applied (e.g. Arctic Council 2013).

## Humans settlements: The North Water as a gateway

In this section, we shall focus on social features that add to the complexity of the human involvement with the North Water. Humans are not simple consumers of the animals on offer, but they also have their own sense of what matters. Clearly, in hard times, of which there have certainly been many, the Inuit have had little choice of diet and life-style. Yet, there have also been preferences and not least practical skills that have placed the social communities in particular relations to the resources, and varying over time (Flora et al. 2018).

Prior to the Thule Culture, several other cultures had left their traces in the North Water area, if not always very conspicuous (Schledermann 1990; Grønnow and Sørensen 2006). The true pioneer settlers in the area arrived from North America about 4500 years ago. Some moved further south and populated West Greenland, others moved northeast and descended into East Greenland. For all of them, the polynya served as a steppingstone (however fluid). The populations in the Northwest remained small, and in a period from 100 bc to 650 ad, both the Thule region and the rest of Greenland were uninhabited.

Around 650 ad, people again moved into the High Arctic and the North Water area. They are known from Inuit myths as the Tunit people and named Late Dorset by archaeologists (Appelt 2004). The technology of the Tunit did not include dog sledges, bows and arrows, and sea-going vessels (Appelt et al. 2016). The large whales of the polynya were not hunted, but faunal materials show that the Tunit were excellent walrus hunters (Gotfredsen et al. 2018). In fact, this period probably saw the most intensive exploitation of the walrus population of North Water before the introduction of modern hunting technology; seals, birds, and foxes also formed the basis of Late Dorset subsistence (Appelt et al. 2016).

The history of settlements in the area becomes complicated in the last century or so of the Late Dorset period. There is archaeological evidence of Norse visitors from the settlements in West Greenland and, at the same time, the Inuit of the early, so-called Ruin Island phase of the Thule Culture reached the polynya (Appelt 2004). Thus, around 1250–1300 ad, the region was the scene of cross-cultural contacts and probably of exchange of items and raw materials such as ivory and meteoric iron (and perhaps genes and diseases) (Hastrup et al. 2018). The Tunit seem to have disappeared around 1300 ad, while the incoming Thule Inuit whalers settled in the entire North Water area and the rest of Greenland during the following century.

The Thule Culture settlement in the area went through complicated developments. Archaeological evidence suggests the co-presence of different Inuit groups—probably resulting from different immigrations from Alaska and Canada during the period of c. 1250–1450 ad. Moreover, excavations of stratified middens show shifts in subsistence economies through time. For instance, the exploitation of bowhead whales ceased during the fifteenth century, possibly owing to shifting migration patterns of the large whales, in turn due to the onset of the Little Ice Age (c. 1350–1800). The earliest Inuit sites are situated next to whale hunting grounds, whereas later sites generally are smaller, more spread out in the region, and aimed at a utilizing a broader spectrum of resources. Only the early sites (Ruin Island group) include *qassiit*—men’s houses—probably connected to the hierarchical organization of Inuit whaling (e.g. Holtved 1944). There is not much archaeological evidence for Inuit settlement around Smith Sound during the sixteenth and early seventeenth century; episodes of isolation and periodical abandonment of the entire area could be the reason why (Schledermann and McCullough 2003). Certainly, from the seventeenth to nineteenth centuries, the Inughuit on the Thule side became isolated from the rest of the world. During this period, important parts of the material culture were lost.

When the Inughuit were ‘rediscovered’ from the south in 1817, they no longer had bows and arrows, fishing leisters and kayaks, and the size of the population was remarkably low. We can follow the development through the nineteenth century via Kane (1856, II: 211), who counted 140 persons in the entire region in the 1850s, and Hayes who counted only a hundred in the early 1860s, “a very considerable diminution since Dr Kane left them, in 1855” (Hayes 1866, p. 386). Part of this decrease was probably owing to epidemics brought by foreigners. Another factor may have been related to the impoverished technologies hampering the hunt. In this light, the arrival of some 16 Baffinlanders—vaguely remembering having heard of kinsfolk on the other side of the Sound from foreign whalers among others—made a remarkable impact (Rasmussen 1908, p. 24; Schledermann and McCullough 2003, p. 125). Knud Rasmussen, who interviewed people in Thule in 1903, talked with one of the immigrants, still alive, who said:

We taught these people many things. We showed them how to build snowhuts with long tunnel passages and an entrance from below…

We taught them to shoot with bow and arrows. Before our arrival they did not shoot the many reindeer that are in their country…

We taught them to spear salmon in the streams. There were a great many salmon in the country, but they did not know the implement that you spear them with.

And we taught them to build kayaks, and to hunt and catch from kayaks. Before that they had only hunted from the ice, and had been obliged during the spring to catch as many seals, walruses and narwhals as they would want for the summer, when the ice had gone…

But we adopted their type of sledge, for it was better than ours, and had uprights on it. (Rasmussen 1908: 32–33)

What transpires here is the magnitude of technological loss that these people had suffered. It also testifies to the sense of being neighbours across the Sound, although relations had been periodically lost.

The Inughuit were perceived to live on the edge of the inhabited world for generations of scholars to come (e.g. Boas 1964 [1888]). What is evident from all ethnographic works since then, including the classical work by Mauss (1979 [1906]) and Rasmussen (1921), is both that the stark seasonal distinction between summer and winter and the seasonal migrations of the animals formatted social life to the core. This also transpires very clearly from the important contribution to ethnography made by Holtved (1967), an archaeologist who spent several years in the region 1935–1937 and 1946–1947. Mobility remained a permanent feature of social life in the region and beyond. Seasonal movement is a key element in their social resilience, as is the power of anticipating the possibilities for hunting and a high degree of flexibility (Hastrup 2009).

Today, the Inughuit live either in the town of Qaanaaq with c. 640 inhabitants, or in one of the three remaining small settlements, inhabited by c. 45, 35, and 20 inhabitants, respectively (Statistics Greenland 2017). The ice still is a major factor in their mobility, also in their moving away from the smaller settlements, not necessarily because there is no game there, but because connections to the rest of the region is hampered by the dwindling sea ice. They still depend on living resources and their seasonal variation—and not least on their own ability to anticipate animal movement (Nuttall 2010; Hastrup 2013).

## Concluding remarks

In this article, we have wanted to highlight the multiple dimensions of the North Water as a site for particular life forms, human and animal. The multi-species sociality of the North Water is not only based on adaptive synchronicity but has an element of chance connections between species at many trophic levels with each their life cycle and temporal dynamics. Human movements are not directly co-variant, but nevertheless deeply implicated with ice edges, sea currents, animal breeding patterns, and climate variations through their joint dependence on the North Water.

The lack of stability, and the degree of chance implied in a positive feedback between the various time scales of the agents in the North Water, is underscored by the present destabilization of the environment, in terms of both the rising temperatures and the increasing pollution of the sea—originating elsewhere. This reminds us that the North Water is not only an independent agent in local history, but it is also deeply affected by global processes and human actions in other regions. The question is when (or if) the particular multi-species sociality created by a reasonably predictable polynya will find itself in a fast transition—from where the North Water community can no longer reproduce itself (Jeppesen et al. 2018).

To answer this huge question, a sustained effort of interdisciplinarity is necessary, given the complex interplay between physical, biological, social and political processes that are conjoined in the transformation of the North Water.
